# The Multifaceted Granulosa Cell Tumours—Myths and Realities: A Review

**DOI:** 10.5402/2012/878635

**Published:** 2012-09-13

**Authors:** Rani Kanthan, Jenna-Lynn Senger, Selliah Kanthan

**Affiliations:** ^1^Department of Pathology and Laboratory Medicine, University of Saskatchewan, Saskatoon, SK, Canada S7N-0W8; ^2^Department of Pathology and Laboratory Medicine, Royal University Hospital, 103 Hospital Drive, Room 2868, G-Wing, Saskatoon, SK, Canada S7N-0W8; ^3^Department of Surgery, University of Saskatchewan, Saskatoon, SK, Canada S7N-0W8

## Abstract

*Background*. Granulosa cell tumors (GCTs), representing ~2% of ovarian tumours, are poorly understood neoplasms with unpredictable and undetermined biological behaviour. *Design*. 5 unusual presentations of GCT and a retrospective 14-year (1997–2011) surgical pathology review based on patient sex, age, tumour type and concurrent pathology findings are presented to discuss the “myths and realities” of GCTs in the context of relevant evidence-based literature. *Results*. The 5 index cases included (1) a 5 month-old boy with a left testicular mass, (2) a 7-day-old neonate with a large complex cystic mass in the abdomen, (3) a 76-year-old woman with an umbilical mass, (4) a 64-year-old woman with a complex solid-cystic pelvic mass, and (5) a 45 year-old woman with an acute abdomen. Pathological analysis confirmed the final diagnosis as (1) juvenile GCT, (2) macrofollicular GCT, (3) recurrent GCT 32 years later, (4) collision tumour: colonic adenocarcinoma and GCT, and (5) ruptured GCT. *Conclusion*. GCT is best considered as an unusual indolent neoplasm of low malignant potential with late recurrences that can arise in the ovaries and testicles in both the young and the old. Multifaceted clinical presentations coupled with the unpredictable biological behaviour with late relapses are diagnostic pitfalls necessitating a high degree of suspicion for accurate clinical and pathological diagnosis.

## 1. Introduction

 Granulosa cell tumours (GCTs) though accounting for approximately 70% of malignant sex-cord stromal tumors are rare, comprising only 2–5% of all ovarian neoplasms [1–3]. These tumours arise from granulosa cells that are hormonally active stromal elements in close association with ovarian oocytes, which are responsible for the production of estradiol [1]. The exact etiology of this malignancy remains unknown, with no identification of specific defined risk factors [3]. The typical clinical scenario of a GCT is an older postmenopausal woman with menstrual abnormalities who is found to have a singular pelvic mass that is curable by en-bloc resection. While such typical cases do exist, characteristics of GCTs in clinical practice do not always fit within the confines of these parameters. As such, seeing past this “myth” to recognize and identify uncommon “realities” is necessary for the accurate diagnosis and management of GCTs.

The aim of this study is to discuss the “myths and realities” of GCTs through a series of indexed cases in the context of relevant evidence-based literature.

## 2. Materials and Methods

 Five patients presented to our hospital with “unusual” GCTs, prompting a complete chart review for each patient. Further, a comprehensive fourteen-year (1997–2011) search using the Laboratory Information System (LIS) identified additional patients with GCTs. Original slides were obtained to verify their histomorphological diagnosis. Patient demographics including age, sex, and tumour site were collected for analysis.

 A literature search using the National Library of Medicine Interface PubMed was conducted using the search terms “granulosa cell” limited to the English language. The bibliographies of these manuscripts further identified relevant secondary sources. 

This study was conducted with ethics approval from the University of Saskatchewan Biomedical Research Ethics Review Committee.

## 3. Results

### 3.1. Case 1: 5-Month-Old Boy with a Left Testicular Mass

**Figure 1:**
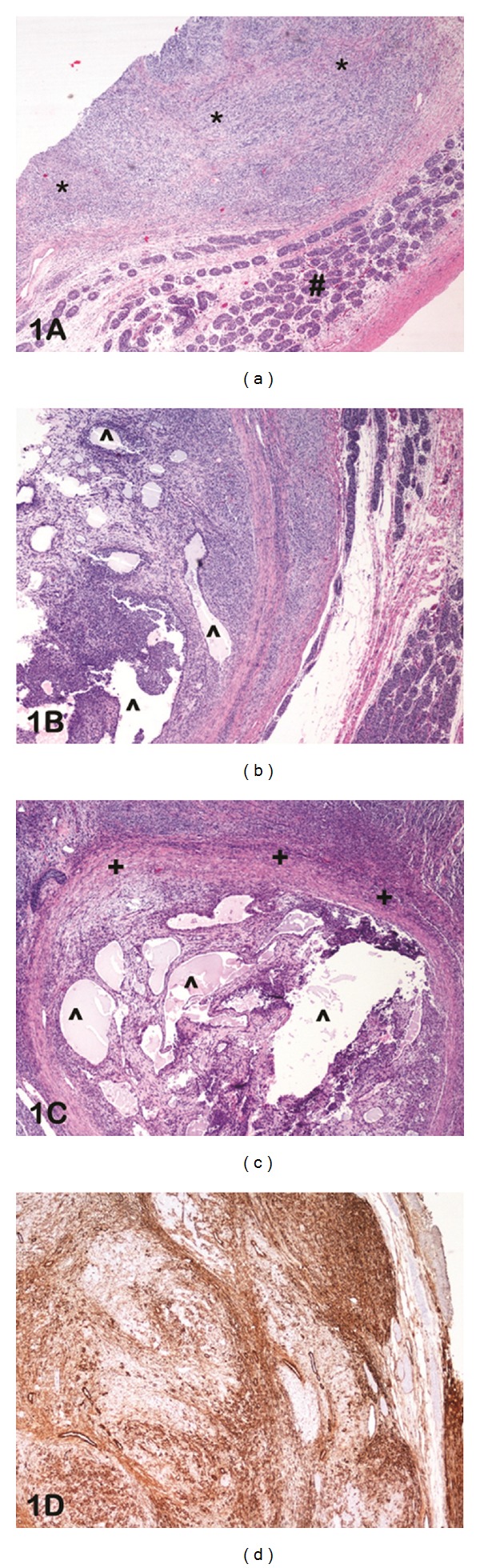
(a) Photomicrograph of haematoxylin and eosin-stained slide at low power (lens objective ×4) shows the presence of solid sheets of neoplastic cells (*) adjacent to normal testis (#). (b) Photomicrograph of haematoxylin and eosin-stained slide at low power (lens objective ×4) shows the presence of cystic spaces (^∧^) within the neoplasm. (c) Photomicrograph of haematoxylin and eosin-stained slide at medium power (lens objective ×10) shows the presence of fibrocollagenous stroma (+) surrounding the solid-cystic (^∧^) neoplasm. (d) Photomicrograph of immunohistochemical staining with inhibin shows diffuse cytoplasmic staining of the lesional cells.

A left scrotal mass was detected in a 5-month-old baby boy. Ultrasonography confirmed the mass to be a 2 cm lesion, and an orchiectomy was carried out. On histological examination, the tumour was solid with regions of cystic spaces (Figures 1(a) and 1(b)). Amphophilic cytoplasm with indistinct cell borders and an extensive fibrocollagenous stroma were identified (Figure 1(c)). The lesional cells were positive to inhibin (Figure 1(d)), confirming the sex-cord stromal nature of this neoplasm. This patient was the only male diagnosed with GCT in our series. Twenty years post-operatively he is doing well, with no evidence of recurrence.

### 3.2. Case 2: 7-Day-Old Neonate with a Large Complex Cystic Mass in the Abdomen

**Figure 2:**
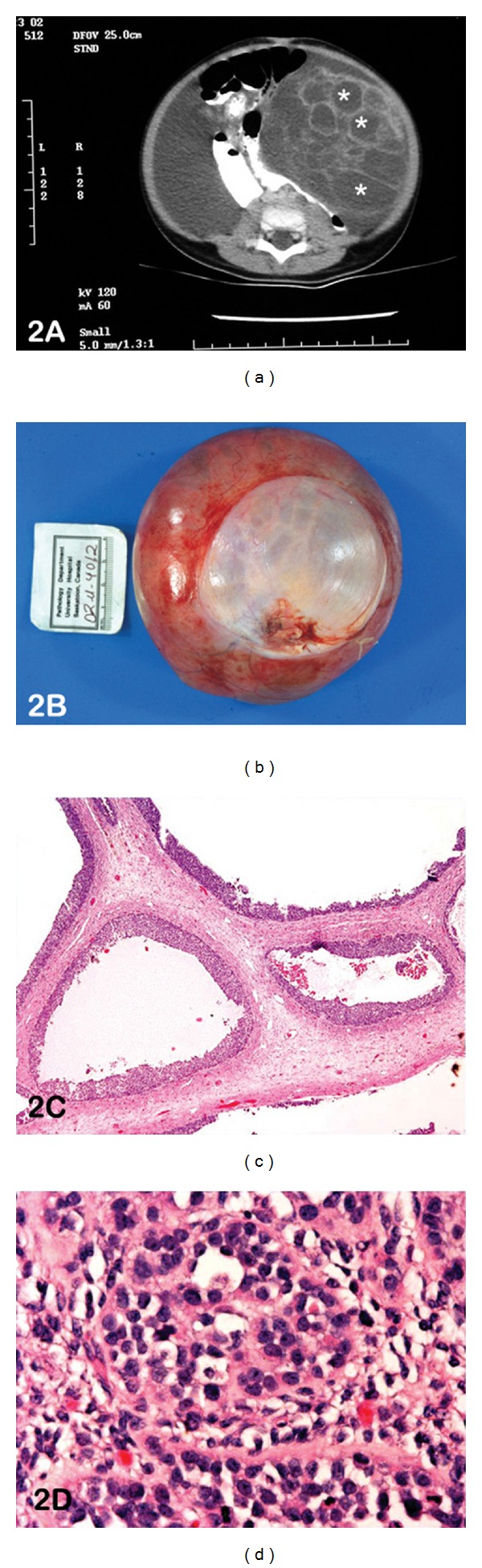
(a) CT scan abdomen shows the presence of 10 cm multi-septated cystic mass in the left quadrant (*). (b) Photograph of the gross specimen confirms a large cystic mass measuring 12 × 11 × 4.5 cm. (c) Photomicrograph of haematoxylin and eosin-stained slide at medium power (lens objective ×10) shows a multicystic lesion lined by granulosa cells. (d) Photomicrograph of haematoxylin and eosin-stained slide at high power (lens objective ×20) shows the presence of neoplastic cells with amphophilic cytoplasm and nongrooved nuclei.

 Seven days after birth, a female neonate developed increased abdominal distension and constipation. A large mass was palpable on abdominal exam that prompted ultrasonography, which detected a 10 cm multiseptated cystic, mass. This was further confirmed by computed tomography (CT) scan, which showed the mass to be surrounded by a large volume of fluid (Figure 2(a)). Hematological investigations showed high levels of estradiol (310), prolactin (34.9), and thyroid-stimulating hormone (TSH, 11.05) with normal levels of luteinizing hormone (LH), follicle-stimulating hormone (FSH), *α*-fetoprotein, and *β*-human chorionic gonadotropin (*β*-HCG). The young girl was taken to the operating room for a laparotomy, which revealed a large cystic mass of the left ovary with torsion (Figure 2(b)). An oophorectomy with preservation of the left fallopian tube was carried out. On follow-up abdominal and pelvic CT, no evidence of residual disease was detected, and estradiol and TSH levels normalized. Eighteen years post-operatively she is doing well, with no evidence of recurrence.

 On histological examination, a multicystic lesion lined by granulosa cells was identified (Figure 2(c)), without the typical theca internal layer lining the cysts. Solid proliferations of granulosa-like cells were recognized in the ovarian stroma, and focal areas with a solid insular pattern and nongrooved nuclei were present (Figure 2(d)). Histological features were between those common to adult GCT (AGCT) and juvenile GCT (JGCT). An almost exclusive macrofollicular pattern typical of AGCT was contrasted by small areas of the solid, insular pattern as seen in JGCT. Cytoplasm was more abundant than generally recognized in AGCT, but less in JGCT. The unusual facet in this case is the clinical presentation of an unsuspected GCT in a seven-day-old neonate.

### 3.3. Case 3: 76-Year-Old Woman with an Umbilical Mass

**Figure 3:**
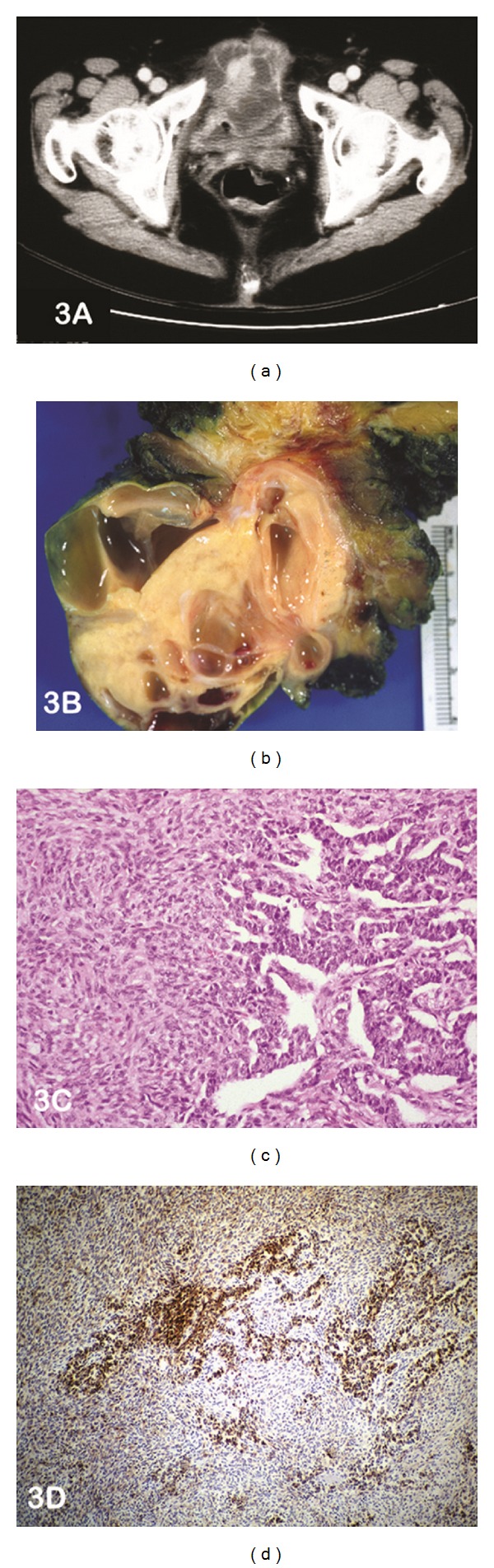
(a) CT scan abdomen shows the presence of a complex mass arising from the dome of the bladder ?urachal carcinoma. (b) Photograph of the cut section of the gross specimen confirms a solid mass with cysts of varying sizes. (c) Photomicrograph of haematoxylin and eosin-stained slide at medium power (lens objective ×10) shows the presence of sheets of spindle cells with focal retiform-like areas. (d) Photomicrograph of immunohistochemical staining shows focal-positive staining to inhibin A.

 A 76-year-old woman presented with a growth at the umbilicus and bowel obstruction. CT scan showed a complex mass arising from the anterior bladder wall/dome of the bladder (Figure 3(a)). With the clinical suspicion of a urachal carcinoma, the patient was taken to the operating room where she underwent a partial cystectomy and anterior abdominal wall mesh reconstruction. The mass was smooth and solid cystic (Figure 3(b)), with extension from the bladder's dome to the umbilicus. At microscopic examination, sheets of spindle cells in focal retiform-like areas with uniform grooved nuclei were identified (Figure 3(c)). Immunohistochemical analysis found the mass to be positive to vimentin, with focal positivity to inhibin A (Figure 3(d)) and cytokeratin. 

### 3.4. Case 4: 64-Year-Old Woman with a Complex Solid-Cystic Pelvic Mass

**Figure 4:**
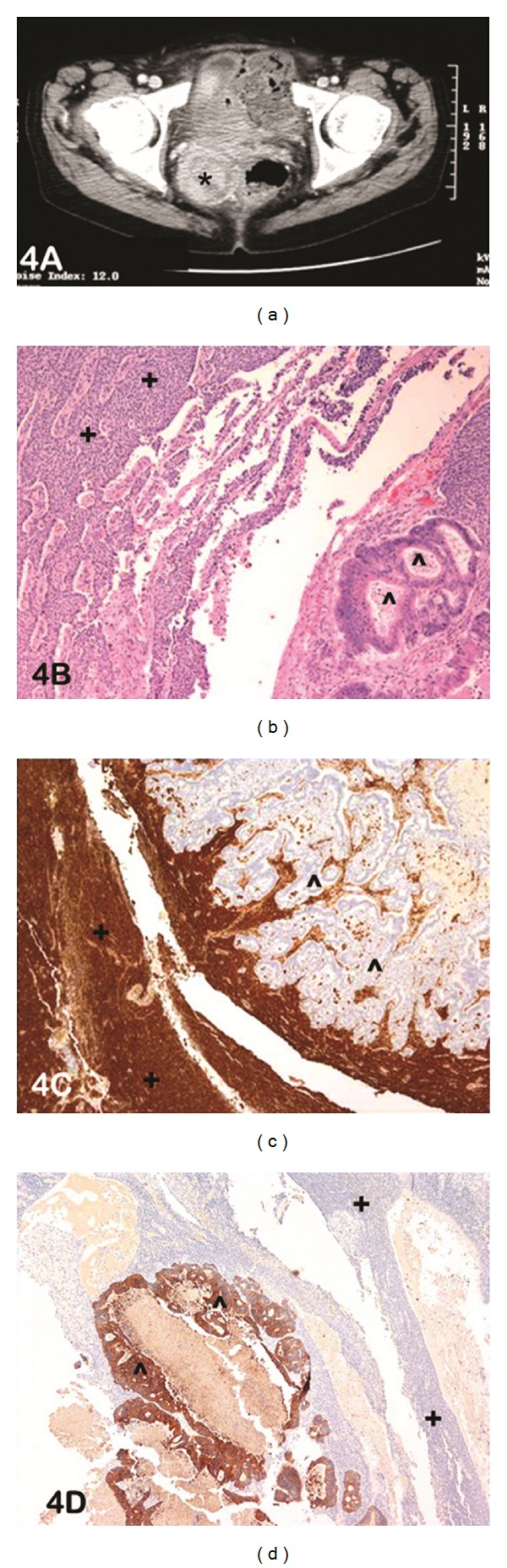
(a) CT scan abdomen shows a large heterogenous pelvic mass (*), solid and cystic. Abscess, ?GIST extracolonic mass, ?Ovarian tumour. (b) Photomicrograph of haematoxylin and eosin-stained slide at low power (lens objective x4) shows the presence of two distinct histological phenotypes: a) diffuse cords of neoplastic cells in the upper left-hand corner (+) and b) neoplastic glands with “dirty” necrosis in the lower right-hand corner (^∧^). (c) Photomicrograph of immunohistochemical staining shows diffuse-positive staining to inhibin A in the solid cords of neoplastic cells (+) with negative staining in the adjacent glandular component (^∧^). (d) Photomicrograph of immunohistochemical staining shows diffuse positive staining to CK20 in the glandular component (^∧^) with negative staining in the adjacent solid cords of neoplastic cells (+).

 An incidental pelvic mass was detected in a 64-year woman with a past history of total abdominal hysterectomy and bilateral salpingooophrectomy for a GCT 16 years prior. A CT scan confirmed the presence of a large heterogenous solid-cystic pelvic mass (Figure 4(a)). The preoperative diagnosis of this mass included abscess, gastrointestinal stromal tumor, and extracolonic mass. She underwent laparotomy with pelvic washings for cytology and en-bloc resection of the left ovarian mass. On gross examination, the mass was a yellow-tan cystic structure with regions of haemorrhage. Microscopically, the tumour was highly cellular, with scant cytoplasm. Multiple histological patterns including focal glandular pattern with “dirty” necrosis reminiscent of colonic adenocarcinoma admixed with diffuse, microfollicular, and cords of neoplastic cells were observed (Figure 4(b)). Regions of edema and cystic change were further identified. Deeper sections confirmed the presence of the glandular neoplasm originating from a diverticular outpouching of the overlying colonic mucosa, confirming the presence of colonic adenocarcinoma arising in a diverticulum of the large bowel. This was further supported by immunohistochemistry that confirmed the two components of this collision tumour with inhibin positivity in the GCT component (Figure 4(c)) and cytokeratin positivity in the adenocarcinoma component (Figure 2(d)). 

### 3.5. Case 5: 45-Year-Old Woman with an Acute Abdomen

 A 45-year-old woman presented to the Emergency Room with a two-year history of menorrhagia and acute dyspnea. An enhanced CT scan revealed a giant mass (estimated size, 36 cm craniocaudal) filling the pelvis and abdomen and causing displacement of the bowel loops. A pulmonary embolus was additionally detected, and she was started on anticoagulants. The patient developed imminent renal failure and her hemoglobin levels continued to drop despite receiving 9 units of blood. It was suspected that her mass was haemorrhaging, and she was taken to the Operating Room. A 35–40 cm, multilobulated, haemorrhagic mass was found in her right ovary, and a total abdominal hysterectomy with bilateral oophorectomy, with en-bloc mass resection and partial omentectomy were carried out. 

**Figure 5:**
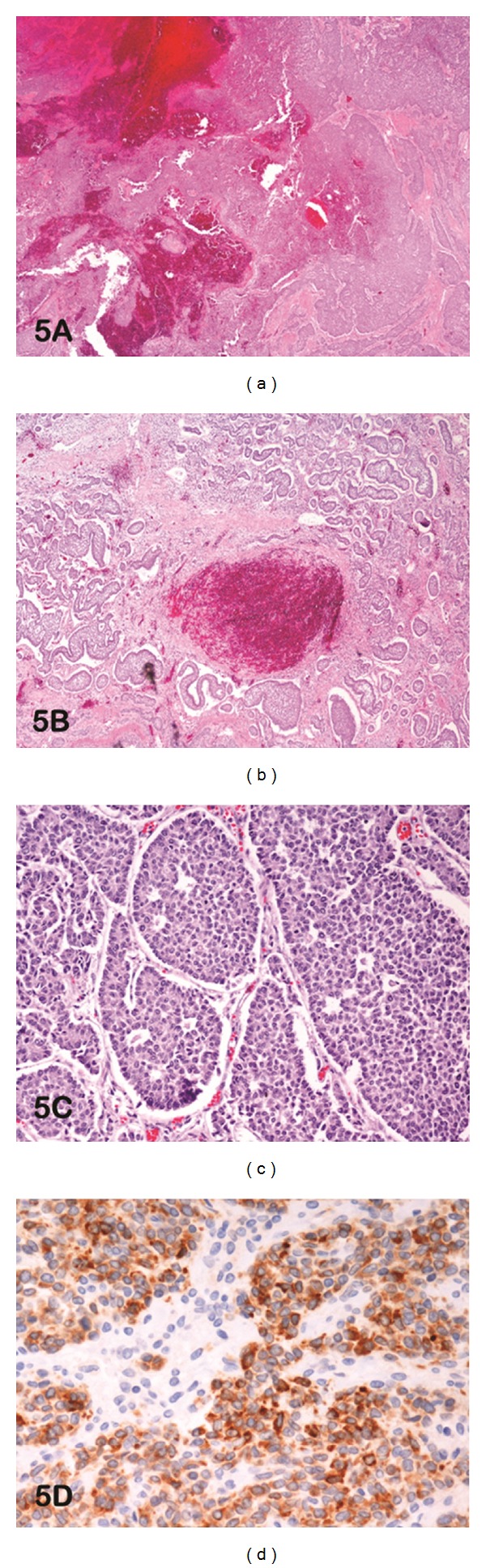
(a) Photomicrograph of haematoxylin and eosin-stained slide at low power (lens objective ×2) shows sheets of neoplastic cells with acute hemorrhage and rupture. (b) Photomicrograph of haematoxylin and eosin-stained slide at low power (lens objective ×4) shows solid nests of primitive granulosa cells with focal hemorrhage. (c) Photomicrograph of haematoxylin and eosin-stained slide at medium power (lens objective x10) shows granulose cells arranged in microfollicles forming Call-Exner bodies. (d) Photomicrograph of immunohistochemical staining shows diffuse-positive cytoplasmic staining to inhibin A.

 Microscopic examination showed the presence of sheets of neoplastic cells interspersed with rupture and acute hemorrhage (Figure 5(a)). The ovarian tumour showed granulosa cells arranged in solid sheets, trabeculae, tubules, and microfollicles that included Call-Exner bodies (Figures 5(b) and 5(c)). Immunohistochemical analysis found the neoplastic cells to stain positive for vimentin, inhibin (Figure 5(d)), CD99, and AFP. The unique presentation of this GCT is an acute abdomen due to tumor rupture with hemorrhage.

**Surgical Review**

A 14-year (1997–2011) surgical pathology review yielded 37 cases of GCT, with an overall prevalence of 0.0041% (37/902 100). Of these cases, 36 were females and one male (Index case 4). Within the female population, patient's age ranged from 7 days to 85 years, with a mean of 52 years (median 48 years). Locations of GCTs included 17 cases in the right ovary (47.2%), 15 in the left ovary (41.7%), and one (2.8%) in each of the bowel, abdominal wall, and omentum. Only four (10.8%) GCTs were of the juvenile type. Concurrent pathologies included leiomyoma of the uterus (21%), benign cysts (14%), adenomyosis (7%), coexisting ovarian adenocarcinoma (3%), and endometrial carcinoma (3%).

## 4. Discussion

 Granulosa cell tumours (GCTs) were first described in 1855 by Rokitansky as chronicled in Chew et al.'s manuscript [4]. GCTs are rare sex-cord stromal tumors that are thought to arise from the normal proliferating granulosa cells of the late preovulatory follicle as they share many morphological and biochemical features with these cells. Additionally, theca cells, interstitial cells, and stromal fibroblasts have also been delegated as cells of origin for GCT. [5]. This probably accounts for their origin when they occur in the testis. However the origin of an adult GCT of the testis remains poorly understood [9]. Recently, there is growing evidence to suggest that granulosa stem cells (GSCs) do exist. Identification of normal and neoplastic GSCs and the factors that regulate their behavior will determine the treatment of all ovarian cancers including GCTs in the future [2]. However, currently, little is known regarding the molecular and genetic changes associated with GCTs. They have been associated with a variety of genetic abnormalities such as trisomy 12/14, monosomy 22, and >90% of adult GCT's have a missense somatic C134W mutation in the *FOXL2* gene [6]. In men, this tumor is usually associated with defects or anomalies of the Y chromosome. 

The typical clinical scenario of a GCT is usually a middle-aged female presenting with a pelvic mass who is cured by en-bloc resection of the mass. Pathological diagnosis is often straightforward in these typical cases and the tumour rarely reoccurs. These characteristics however are not generalizable to all GCTs as these lesions can present in many different facets and have a tendency to behave unpredictably, thus complicating both diagnosis and therapeutic management. Accurate recognition of the true “realities” of multifaceted GCT is therefore vital for the precise diagnosis and management of these lesions. Some of the existing myths of GCTs will now be contrasted with their true realities in the context of evidence-based literature.

### 4.1. Myth 1: GCT Is a Benign Tumour

#### 4.1.1. Reality: GCT Is a Low-Grade Malignant Tumour

Ovarian cancer is the second most common type of gynecological malignancy [1]. This cancer can be divided into three types based on the cell of origin (germ, epithelial, and stromal) with each conferring different histopathological features and clinical outcomes. Stromal tumours are further classified based on the tissue types involved as Sertoli, Leydig, theca, and granulosa. Granulosa cell tumours (GCTs) account for 1-2% of all ovarian tumours [7] and arise from the granulosa cells that normally surround the oocytes and line the developing follicle. Two theories exist to explain the exact etiology of these tumors. These include a) these neoplasms are derived from the mesenchyme of the developing genital ridge and b) these neoplasms arise from precursors within the mesonephric and coelomic epithelium. The presence of extraovarian GCT's as seen in our case 3 supports the latter theory. To date, however, no definite aetiologies or risk factors have been identified for GCT. Though chromosomal anomalies and/or autocrine and endocrine signalling abnormalities are proposed aetiologies, the current etiology postulated is one of multifactorial origin. These lesions are considered a low-grade type malignancy, with 70–90% of neoplasms being diagnosed at Stage 1 [1]. The high detection rate at an early stage may be due to the endocrine symptoms that often present early in the functioning tumors. Low staging at diagnosis confers an excellent prognosis, with 5-year survival rates reported between 75–95% (Stage 1); however, these rates drop to 55–75% and 22–50% for stages II and III/IV respectively [1]. This may be partially due to limited treatment options for advanced and recurrent disease [6].

### 4.2. Myth 2: GCT Only Occurs in Females

#### 4.2.1. Reality: GCT Also Occurs in Males

Although predominantly occurring in the granulosa cells of the female ovary, GCTs are also reported to arise within the male testis, as seen in our index case 1. Testicular sex-cord stromal tumours are rare, comprising only 4% of all testicular tumours [8]. Juvenile GCT (JGCT) is far more common than adult GCT (AGCT) within the testicle with no preferred laterality within the testis [9, 10]. Approximately half of testicular JGCTs are diagnosed within the first month of life, and over 95% within the first year as seen in our case 1 [11]. The differential diagnosis of testicular JGCT includes yolk sac tumour, undifferentiated sex-cord stromal tumour, gynandroblastoma, and gonadoblastoma [12]. Typically, males present with a painless indolent testicular swelling. Due to estrogen hypersecretion, patients may be impotent, and 25% have gynaecomastia [9, 13]. An intra-abdominal mass of an undescended testis and/or a testicular torsion may additionally be present [11]. JGCTs in undescended testis are benign and do not reach an adequate size to cause pressure on other organs [14]. AGCTs are extremely rare testicular tumors [9, 15–17]. Though patients with lymph node metastases usually have a longer survival period, the presence of distant metastases is usually associated with a dismal prognosis. Initial treatment for testicular GCT is a radical orchiectomy [9]. Diagnosis is often made only by microscopic evaluation. Histologically, testicular GCT resembles ovarian, presenting as a solid, cystic mass with microfollicular, gyriform, insular, and trabecular patterns [13]. Granulosa cells must be present for the diagnosis of GCT [8]. Cells are typically immunopositive for vimentin, inhibin, smooth muscle actin, CD99, and S-100 [9, 18]. Genetically, chromosomal abnormalities such as an atypical Y chromosome and mosaicism may be present [19]. Stage-matched testicular GCT confers a better prognosis than its ovarian counterpart [13]. 

### 4.3. Myth 3: GCT Is a Tumour of Middle-Aged, Postmenopausal Women

#### 4.3.1. Reality: GCT Occurs in Patients of a Wide-Age Range

Most patients with GCTs are perimenopausal or early postmenopausal, with a median age of diagnosis between 50–54 years [1]. Nevertheless, as our surgical review demonstrates, GCTs may arise in neonates (index case 2) or in patients over the age of 80 (series). Thus GCTs can occur at any age [20]. GCTs in neonates, as in index 2, are a rare occurrence, with few reported cases in less than one year of age. Childhood ovarian juvenile-type GCT are also rarely reported [21]. A common misconception is that the two subclassifications of GCTs, adult-type GCT (AGCT) and juvenile-type GCT (JGCT), refer to the age of development. Though AGCT and JGCT occur more often in adults and children, respectively, either form may present throughout the entire population. The majority (95%) are AGCTs, more commonly seen in adults [22, 23]. The two forms differ with regard to histologic features and clinical behaviour [24]. AGCT has been reported in children [25]; however, less than 1% of these lesions occur in prepubertal girls [26]. Upto 90% of JGCTs are diagnosed in patients under the age of 30 with half of JGCT cases seen in less than 10 years of age [22, 27] and 10% occurring in infants less than one year [5]. Occasionally it can occur in pregnant women [22]. 

Differences between AGCT and JGCT are distinct; therefore, accurate identification is critical to guide patient management. Unlike AGCT, JGCT is considered by many to be a relatively benign tumour and in infantile males is the common type of sex-cord stromal tumour of the testis [28]. Cells producing hormones such as estradiol are present in 70% of JGCTs [29]. As such, in young patients, clinical evidence of isosexual precocious pseudo-puberty including breast enlargement, pubic and axillary hair development, vaginal secretions, irregular uterine bleeding, advanced somatic/skeletal developments, and secondary sex characteristics may all be associated with JGCT [2, 5]. Recognition of these clinical findings may be central to the accurate diagnosis of GCT. These patients often have elevated estradiol levels, though this is not a requirement for precocious puberty. The risk of precocious puberty is especially high in JGCT patients under one year of age, but GCT in this age range is rare [30]. Such symptoms are present in 80–90% of patients under the age of 8 with JGCT [27]. Postpubertal patients may experience abdominal pain, swelling, menstrual irregularities, and/or amenorrhea [2]. On radiologic imaging, JGCT is often indistinguishable from other ovarian neoplasms [30], and identification of histopathology features such as a nodular/diffuse cellular growth and/or macrofollicules with eosinophilic/basophilic fluid in their lumen is often required for correct diagnosis. As these are younger women, uterine sparing surgery with conservation of the contralateral tube and ovary is recommended at the outset for maintenance of fertility. Advanced-stage disease is responsive to combination chemotherapy with platinum agents [5, 6]. Advanced-stage JGCT can be aggressive with a short interval to relapse [2]. The overall prognosis for JGCT is excellent, with reports of survival as high as 97% in one study with a 3.5-year followup [5]. After-resection, gross examination of a JGCT is similar to that of an AGCT. On average, tumours measure 12 cm and are solid, but may have cystic regions [27]. Microscopically, these tumours are solid/cellular, with follicle formation, edema, and loose stroma. Hyperchromatic granulosa cells with rounded nuclei surrounded by eosinophilic or vacuolated cytoplasm and high mitotic rates are additionally seen [2, 5]. Unlike AGCT, few Call-Exner bodies are identified [5].

### 4.4. Myth 4: GCT Presents As a Mass Lesion

#### 4.4.1. Reality: Presentation Is Diverse

Two-thirds of GCT patients present with endocrine syndromes due to functional tumours [31]. Estradiol is one of the first hormones to be secreted by GCTs and is responsible for clinical manifestations [3]. Among females, symptoms are dependent on the reproductive stage and type of tumor secretion. Pre-pubescent girls may experience isosexual precocious puberty as a result of increased estrogen levels caused by hyperestrogenism [1]. Though hyperestrogenism is the common form of endocrine abnormality in GCTs, it is important to consider juvenile GCT in females presenting with androgen excess and precocious puberty [5]. The adult GCT appears to be the most common type of GCT associated with virilisation, suggesting a propensity for increased androgen secretion in AGCTs [32]. By contrast, these elevated estrogen levels in adults can cause abnormal uterine bleeding, menstrual irregularities, menorrhagia, or amenorrhea [20, 27]. Patients with virilising GCTs can present with hirsutism, clitoromegaly, increased abdominal size, amenorrhea, and deepening of voice [26]. Rare case reports document JGCTs presenting with paraneoplastic syndrome of hypercalcemia [33] and Meig's syndrome of pleural effusion with ascites [29]. Adult patients may present with vaginal bleeding caused by endometrial hyperplasia or uterine cancer as a result of prolonged exposure to tumor-derived estrogen. In addition, GCT is a vascular tumor that may occasionally rupture and result in abdominal pain, hemoperitoneum, and hypotension, mimicking an ectopic pregnancy in younger patients. Tumor rupture is often attributed to hemorrhagic cysts in upto 10–15% of the cases [2, 27]. Unusual presenting symptoms of isolated synchronous breast metastases from GCT have also been reported [34]. Occasionally GCTs and theca cell tumours have been found in ovaries which show no enlargement and are therefore clinically occult [35, 36].

GCTs are the most common estrogen-producing neoplasms in females and are found to produce estradiol in approximately 40–60% of patients. This estradiol production is directly related to the release of testosterone secreted by the theca cells. However, not all GCTs are hormonally active or have theca cells that secrete testosterone, and therefore diagnostic testing for these hormones lacks sensitivity and specificity. Normal granulosa cells are responsible for production not only of estradiol but also peptide hormones including inhibin, activin, follistatin, and antimullerian hormones [25]. GCT patients usually present with elevated levels of inhibin, a negative feedback regulator of FSH secretion; however, this hormone is not specific for these tumours [2, 3, 37]. Mom et al. evaluated the sensitivities and specificities of serum inhibin levels in 30 women with granulosa cell tumors (inhibin A was 67 and 100% and inhibin B was 89 and 100%, resp.). Serum inhibin levels are currently available for diagnosis and clinical followup of women with granulosa cell tumors of the ovary [38] It can, therefore be used to monitor response to therapy or to detect recurrences. Excessive inhibin secretion may cause secondary amenorrhea in patients with GCTs [37]. 

Mullerian inhibitory substance (MIS) which is produced in the developing follicles is often elevated in GCTs, though it is not specific for diagnosis [2, 3]. This hormone is produced exclusively by granulosa cells in postnatal females and both prenatally and postnatally by the Sertoli cells in the male testis. This hormone functions in male fetuses to induce regression of the mullerian system. Normally, MIS is found in low levels in reproductive-aged females and functions as a paracrine inhibitory factor that decreases the response of the resting ovarian follicle to follicle-stimulating hormone (FSH) thus ensuring the emergence of a single dominant follicle. Serum MIS may be a marker of ovarian reserve and typically disappears from the serum after menopause or bilateral oophorectomy. However, in patients with GCTs, levels have been shown to parallel the extent of disease. Serum MIS levels are not routinely available for clinical use in the context of GCT diagnosis and followup. However, a commercial version of the ultrasensitive ELISA assay has become available and may lead to wider clinical use of MIS in the future [39]. Serum MIS levels thus correlate well with tumor presence in patients with GCTs and elevated levels are considered highly specific for GCT in postmenopausal or oophorectomized women. It may also be elevated in women with Sertoli-Leydig cell tumors of the ovary, but is not typically produced by other gonadal or extragonadal tumors. This is in sharp contrast to inhibin and estradiol levels, both of which may be elevated in a variety of other extraovarian disorders. This marker may thus eventually be used for both diagnosis and follow-up evaluations of patients with GCTs. Preclinical research is also ongoing to evaluate the clinical use of targeting the MIS receptor for therapy in cancers expressing this receptor.

Follicle regulatory protein is secreted by the granulosa cells and is elevated in some patients with GCTs. However, the clinical significance of this marker is still undetermined [20, 27]. 

### 4.5. Myth 5: GCT Lives Alone

#### 4.5.1. Reality: GCT Can Be a “Symbiotic Parasite”

Primary synchronous malignancies are rare, with an incidence of 1–6% among cancer patients. The most common pair of synchronous lesions involves the endometrium and the ovary [40]. GCTs have been reported in coexistence with a number of pathologies including mucinous cystadenoma [7, 41, 42], cystic teratoma [7], ovarian fibroma [43], ovarian angiosarcoma, adenosarcoma, cystadenosarcoma [44], sclerosing peritonitis [45], gastric signet-ring cell carcinoma [46], and cervical lipoleiomyoma [47]. In the indexed case 4 GCT was found to coexist with colonic adenocarcinoma as a unique collision tumor which has been discussed previously in detail [48]. The presence of such coexiting pathologies may contribute to increased confusion and be a deterrent to the accurate clinical and pathological recognition of this uncommon neoplasm. 

Uterine pathologies that have been reported to occur with GCT include glandular hyperplasia, atypical adenomatous hyperplasia, adenocarcinoma insitu, and invasive carcinoma [3]. Endometrial hyperplasia is a common finding alongside GCT, occurring in 25–50%, which may occur due to estrogen produced from the GCT stimulating the endometrium [27]. A simultaneous uterine carcinoma can be found in 5–10% of patients with GCT [31] and are often well differentiated and in an early stage. Perhaps due to the diagnosis at an earlier stage, patients with synchronous endometrial and ovarian cancers have a better prognosis than patients with a single malignancy that is typically detected once it has become more extensive [40].

### 4.6. Myth 6: En-Bloc Resection Alone Cures GCT

#### 4.6.1. Reality: GCT Treatment Is Multimodal and Varies by Stage

 The rarity of these lesions prevents randomized control trials to determine the specific best consensus practice guidelines for the management of GCT. No standard management protocols exist for the management of recurrent GCT. The mainstay treatment for GCT is the same as for epithelial ovarian cancer, that is, surgical excision [6, 20, 27]. Diagnostic laparoscopy has been described for the identification of tumour origin, extent, and resectability; however, currently, laparoscopic resection is not advocated for GCTs. Recommended management for Stage I GCTs differs depending on the patient's age. As the incidence of bilateral disease is quite low, for women with reproductive function less than 40 years old and of reproductive age, fertility sparing surgery of unilateral salpingooophrectomy with endometrial biopsy is recommended, while women under 40 without reproductive function and those over 40 require a total abdominal hysterectomy (TAH) as well as a bilateral salpingooophrectomy (BSO) [3, 20, 27]. In patients with more advanced disease, TAH and BSO with complete tumour debulking are suggested [31]. Improved survival with palliative debulking hepatectomy for an unusual case of a grade I, Stage I granulosa cell tumor that recurred 21 years following initial surgery has also been reported [49]. Peritoneal exploration, washing cytology, peritoneal biopsy, and partial omentectomy have been suggested as part of the staging procedure in all GCT patients. Careful examination of the contralateral ovary and tube, intra-abdominal organs, and peritoneum with sampling of the pelvic and para-aortic lymph nodes are recommended [29]. Aside from being a treatment option, surgery is also necessary for staging and accurate tissue diagnosis [3, 27]. These parameters are important to determine, as poor prognostic features include a tumour size greater than 10–15 cm, a high mitotic index, tumour rupture, and lymphatic invasion [3, 6, 31, 50]. 

 Three forms of adjuvant therapy have been suggested to use in combination with surgery: hormonal therapy, radiotherapy, and chemotherapy. Hormonal therapy is believed to act directly by affecting the tumour and/or indirectly by suppressing gonadotropins or endogenous steroids [51]. Aromatase inhibitors such as anastrozole and letrozole inhibit the conversion of androstenedione to estrone, and estradiol and testosterone to estradiol, reducing aromatization of androgens by upto 90% thereby enhancing the treatment of GCT. Gonadotropin-releasing hormone (GnRH) analogs like leuprolide have been used to decrease stimulation of granulosa cells through inhibition of ovarian steroidogenesis in recurrent GCT. However, the fact that not all GCTs respond to hormonal therapy despite nearly all GCTs containing progesterone receptors indicates that multiple factors play a role in the hormonal regulation of the tumor cell. [51, 52]. Radiotherapy may be used as an adjuvant therapy or in the instance of recurrence, and is associated with an improved survival [27, 29, 52]. Additionally, the use of palliative radiotherapy as an alternative strategy with potential disease control has been useful in symptomatic patients with localized or metastatic disease unqualified for surgery [2, 20]. The use of chemotherapy has yielded encouraging results, associated with a longer disease-free survival [50]. The chemotherapeutic agent cisplatin has the highest reported activity in the ovary, and when combined with doxorubicin, cyclophosphamide, bleomycin, vinblastine, or etoposide, an overall response rate of 60–83% has been reported [31]. The current standard recommended chemotherapeutic regimen for advanced, recurrent, or metastatic GCT is bleomycin, etoposide, and cisplatin (BEP) [3, 20, 27]. Targeted therapy using antiangiogenic agents such as bevacizumab is currently under investigation for GCT [52]. Identification of targets for novel therapeutic agents is also predicted in the future with increased knowledge about the molecular biology of both the normal and neoplastic GSCs [2, 6, 52]. 

### 4.7. Myth 7: GCT Is an Easy Pathological Diagnosis

#### 4.7.1. Reality: GCT Can Mimic Other Pathologies

GCTs are generally large, smooth, or lobulated tumours [53]. On gross examination, the cut surface is primarily solid, with haemorrhagic regions and a gray/white or yellow colour depending on the lipid content [7]. Though haemorrhages may be present in larger tumours, necrosis is rare. A minority of GCTs are partially or completely cystic [53]. These tumours are often filled with serous fluid or clotted blood and may be mistaken for mucinous cystadenoma or cystadenocarcinoma [27]. Pathological examination is still the gold standard to confirm the diagnosis of GCT.

GCTs are sex-cord stromal neoplasms that on microscopic examination contain sex-cord-derived epithelial elements admixed with mesenchymal elements with a variety of combinations and degrees of differentiation [15]. Fibroblasts, granulosa, and theca cells make up a GCT [7]. Depending on 4 variables including age at diagnosis, histology, therapy, and prognosis, GCTs are divided into adult GCTs and juvenile GCT [54]. On histological examination, cells are usually arranged around a central cavity named a Call-Exner body that has a microfollicular growth pattern similar to primordial follicles and contains eosinophilic materials as well as nuclear debris [2]. Call-Exner bodies are present in 30–60% of AGCTs [1]. A wide variety of growth patterns have been identified, and may be divided into two categories. The well-differentiated type includes microfollicular, macrofollicular, trabecular, insular, solid tubular, and gyriform architectural patterns. The moderately differentiated type includes a diffuse, “sarcomatoid” growth pattern that is easily mistaken for a carcinoma or adenocarcinoma [27] as histological mimics. Nuclear characteristics are a hallmark feature of AGCT, including a uniform, pale, and grooved “coffee bean” shape. These nuclear features may be used to differentiate AGCT with a diffuse pattern from poorly differentiated carcinoma, as carcinomatous nuclei are hyperchromatic and not grooved, and additionally do not demonstrate nuclear atypia and multiple mitotic figures to the same extent [1].

 Immunohistochemical analysis can be used to confirm the diagnosis of GCTs if the lesion's morphology is non-predictive of histogenesis. A study by Nofech-Mozes et al. recently described the concordant immunohistochemical characteristics of primary and recurrent GCTs. Inhibin, calretinin, CD56, and CD99 are part of the immunoprofile for both types of GCT; however, the lack of a single specific marker necessitates a panel of antibodies for the detection of these lesions [55]. GCT cells usually stain positive for inhibin, calretinin, CD99, CD56, vimentin, estrogen and progesterone receptors. Other markers that can be positive leading to diagnostic confusion include CAM5.2, AE1/AE3, CD10, S100, WT-1, smooth muscle actin, and desmin. However, GCT's are usually negative for cytokeratin 7 and epithelial membrane antigen (EMA). The absence of staining with EMA has diagnostic value in distinguishing GCT from its multiplicity of histological look-alikes such as metastatic or primary carcinoma [56].

### 4.8. Myth 8: GCT Rarely Recurs

#### 4.8.1. Reality: GCT Can Metastasize and Recur

GCTs are unpredictable neoplasms that have the ability to extend locally or spread by lymphatics, especially to the para-aortic lymph nodes. Alternatively, dissemination may occur through hematogenous spread, as evidenced by parenchymal involvement [4]. Distant metastatic sites of GCT most commonly include the lung, liver, and brain [34]. 

Recurrent disease tends to occur many years after the initial diagnosis. A quarter of GCT patients will have recurrences, and the mean time to their detection is 5–10 years [3, 27]. 10–20% of patients may develop recurrences as late as twenty to forty years after the primary diagnosis [57, 58]. One-third (33%) of GCTs recur in less than 5 years, half (50%) between 5–9 years, and 17% ten or more years after the initial diagnosis [49, 59]. Splenic rupture from metastatic GCT 29 years after the original curative resection has also been reported [4]. As such, lifelong surveillance for these neoplasms is recommended. Frequent sites of recurrence include the upper abdomen (55–70%) and the pelvis (30–45%) [2]. This suggests that recurrences in early-stage patients may be attributed to preexisting diseased peritoneum during the initial surgery [60]. In early-stage patients, risk factors for relapse include large tumour size, high mitotic index, and tumour rupture; therefore, these features may indicate the need for postoperative adjuvant chemotherapy [27]. Additional postsurgical risk factors include advanced stage of presentation, lymphovascular space invasion, bilaterality, and Ki67/p53 overexpression [60]. Higher stage disease is also related to aggressive tumour behaviour with recurrences [36]. 

The overall ten-year survival rates in patients with GCT range between 60 to 90% [27]. Approximately 80% of females with advanced GCT die due to the disease, which is partly related to the tendency for delayed recurrence [61]. This unpredictability of the time interval for recurrent and/or metastatic disease indicates the requirement for a long-term clinical followup in all cases [3, 27]. 

## 5. Conclusion

 GCT is best considered an unusual indolent neoplasm of low malignant potential with late recurrences that can arise in the ovaries and testicles in both the young and the old. The multifaceted clinical presentations coupled with the unpredictable biological behaviour with late relapses are diagnostic pitfalls necessitating a high degree of suspicion for accurate clinical and pathological diagnosis. Surgery continues to be the primary cornerstone of initial treatment with chemotherapy and/or radiotherapy being reserved for advanced or recurrent disease states. Lack of evidence-based predictive and prognostic factors continues to be a deterrent in accurately predicting the biological behaviour of individual GCTs. However, long-term lifelong followup including physical/pelvic exam, abdominal/pelvic CT scan, and/or tumor markers as available is recommended in all patients with GCTs as delayed tumor recurrences beyond 5 years are characteristic of this disease.
